# A Bibliometric analysis of folate receptor research

**DOI:** 10.1186/s12885-020-07607-5

**Published:** 2020-11-16

**Authors:** Cari A. Didion, Walter A. Henne

**Affiliations:** Governors State University, 1 University Parkway, University Park, IL 60484 USA

**Keywords:** Folate receptor, Folate binding protein, Cancer, Oncology, Macrophage, Imaging, Bibliometrics, Scientometrics, Librarianship-health sciences

## Abstract

**Background:**

The objective of this study was to conduct a bibliometric analysis of the entire field of folate receptor research. Folate receptor is expressed on a wide variety of cancers and certain immune cells.

**Methods:**

A Web of Science search was performed on folate receptor or folate binding protein (1969-to June 28, 2019). The following information was examined: publications per year, overall citations, top 10 authors, top 10 institutions, top 10 cited articles, top 10 countries, co-author collaborations and key areas of research.

**Results:**

In total, 3248 documents for folate receptor or folate binding protein were retrieved for the study years outlined in the methods section search query. The range was 1 per year in 1969 to 264 for the last full year studied (2018). A total of 123,720 citations for the 3248 documents retrieved represented a mean citation rate per article of 38.09 and range of 1667 citations (range 0 to 1667). Researchers in 71 countries authored publications analyzed in this study. The US was the leader in publications and had the highest ranking institution. The top 10 articles have been cited 7270 times during the time frame of this study. The top cited article had an average citation rate of 110 citations per year. Network maps revealed considerable co-authorship among several of the top 10 authors.

**Conclusion:**

Our study presents several important insights into the features and impact of folate receptor research. To our knowledge, this is the first bibliometric analysis of folate receptor.

## Background

As a vital nutrient for normal cell metabolism, folate uptake in the cell occurs via a low-affinity (K_d_ ~ 1–5 μM) transport protein termed the reduced folate carrier [1] and a high-affinity (K_d_ ~ 100pM) cell surface receptor termed the folate receptor (FR) or folate binding protein (FBP) [1–6]. Notably, FR is over-expressed at significant levels in cancer cells and immune cells (e.g., macrophages) where it mediates uptake of folate by receptor-mediated endocytosis [2, 7–12]. Although folate uptake occurs via the reduced folate carrier in virtually all cells of the body, only folate-linked conjugates can enter cells by means of the high affinity folate receptor [7–13].

The folate receptor exists as a family of proteins with three primary forms: FR-α (folate receptor 1) [14], FR-β (folate receptor 2) [15], and FR-γ (folate receptor 3) [16], and folate receptor delta (folate receptor 4) [17]. These folate receptor homologues are related by ~ 70% amino acid sequence identity [5]. FR-α and FR-β are attached to cell surfaces by a glycosylphosphatidylinositol (GPI) anchor, while the rarely expressed FR-γ is hypothesized to be secreted due to lack of a signal for GPI modification [16]. Folate receptor delta does not bind folate and functions at the initial step of oocyte fertilization [18]. In general, FR-α is upregulated in malignant tissues of epithelial origin such as ovarian carcinoma [19–21] while FR-β is overexpressed in certain subsets of macrophages [19].

This prevalence of FR over-expression in numerous neoplasms and macrophage associated diseases, has led to expansive growth in the use of the cognate folate moiety (as well as anti-FR antibodies) to selectively deliver both diagnostic and therapeutic agents. For example, folate has been conjugated to i) protein toxins [13, 22, 23], ii) low molecular weight chemotherapeutic agents [11, 24, 25], iii) MRI contrast agents [26], iv) genes [27–32], v) viral vectors [33, 34], vi) antisense oligonucleotides [35–38], vii) ribozymes [39, 40], viii) radioimaging agents [10, 41–45], ix) liposomes with entrapped drugs [32, 46–48], x) neutron activation complexes [49, 50], xi) immunotherapeutic agents [51–56], enzyme constructs for prodrug therapy [57], nanoparticles [58], drug-linked polymers [59–61], micelles [62], and optical imaging agents [63, 64]. Significantly, the above folate conjugates neither bind to nor transit through the reduced folate carrier: therefore, they exhibit no affinity for most normal cells [65]. Several promising agents have progressed from phase I through phase III clinical trials and the first diagnostic agents could reach the market in the next few years [66].

According to Andres [67], the term “bibliometrics” was first coined in 1969 and described as a means to apply a mathematical and statistical approach to the study of scientific literature. The term is synonymous with “scientometrics.” However, for the purposes of this study we will use the term bibliometrics. As Kotepui, et al. [68], describe, bibliometric studies are used to obtain a research assessment rich with data that support a specific research interest. The data can be used to present a rich visualization about research undertakings the world round. Trends within a particular field are highlighted through descriptive analysis. Productivity can be demonstrated through the number of articles published, an author count, and by institutions or countries of origin among a myriad of other factors. Given the expansive rise in folate receptor related reports over the past several decades, we sought to analyze the entire field of FR literature. To our knowledge, this is the first bibliometric analysis of folate receptor.

## Methods

For this bibliometric study the search of the available literature was conducted using the online index Web of Science by Clarivate Analytics. The Web of Science Core Collection provides regional citation indexes, patent data, specialized subject indexes, and an index of research data sets from within over 33,000 journals [69]. Using the Web of Science Core Collection, limiting for “articles,” four searches were conducted as follows: “folate receptor” or “folate binding protein;” “folate receptor” or “folate binding protein” and “cancer” or “neoplasm;” “folate receptor” or “folate binding protein” and “inflammation” or “macrophage;” and “folate receptor” or “folate binding protein” and “imaging” or “diagnostics.” An Excel spreadsheet was used to collect the following data for each of the four searches: total journal article search results, publication years (range), first known publication, year of publication, top ten countries/regions, top ten organizations, top ten authors, top ten citations, total citations and citations per year. Graphs for these data were performed using GraphPad Prism. VOSviewer version 1.6.11 software [70] was used to create the author co-authorship bibliometric network map (type of analysis = co-authorship, units of analysis = authors, counting method = full counting, minimum number of documents of an author = 10). Bibliometric key topic maps were constructed using VOSviewer version 1.6.11 [70] for both cancer and imaging search schemes (type of analysis = density visualization, item density, minimum number of occurrences = 50 for cancer and 25 for imaging). Based on the number of articles retrieved, we focused primarily on the first search term “folate receptor” or “folate binding protein” leaving more specific analyses for future work of the rich subfields.

## Results

### Literature retrieved for Folate receptor articles

A total of 3248 documents for folate receptor or folate binding protein were retrieved for the study years outlined in the methods section search query (1969-to June 28, 2019). In this study only research articles in English were used for further analysis since English was the dominant language (99.4%) identified. Review articles, conference proceedings, book chapters, etc., were excluded from the search criteria to avoid citation bias and potential duplication of work. A summary of document types found in the search are presented in Table 1.

**Table 1:** Types of retrieved documents for folate receptor research

Rank	Type of Documents	Frequency ***N*** = 4453	Percentage
1	Article	3289	73.86%
2	Meeting Abstract	465	10.44%
3	Review	426	9.57%
4	Proceeding Paper	129	2.90%
5	Book Chapter	89	2.00%
6	Editorial Material	32	0.72%
7	Correction	11	0.25%
8	Letter	7	0.16%
9	News Item	5	0.11%

### Publication and citation growth for Folate receptor articles

**Fig. 1:**
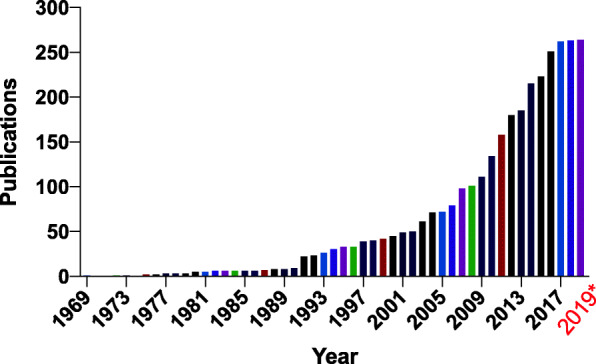
Total number of folate receptor publications per year since 1969-present. *Note: partial year

There was a dramatic increase in the number of folate receptor works during the study period (Fig. 1). The range was 1 per year in 1969 to 264 for the last full year studied (2018). The highest productivity to date (263 publications) was in 2018: however, the number of articles generated per year appears may have started reaching a plateau in 2015. A total of 123,720 citations for the 3248 documents retrieved represented a mean citation rate per article of 38.09 and range of 1667 citations (range 0 to 1667).

### Publications by country for Folate receptor articles

**Fig. 2:**
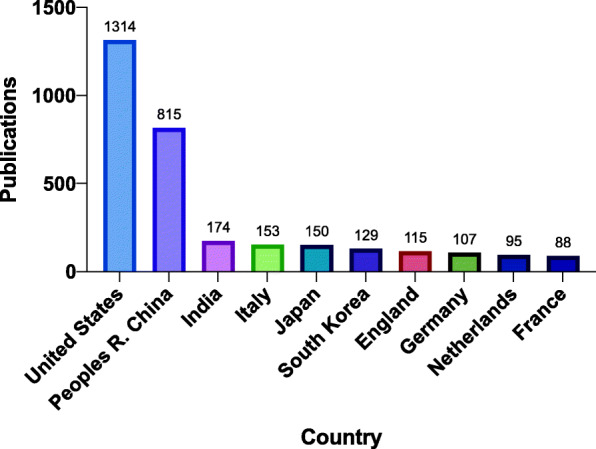
Publications for the top 10 countries in folate receptor research

Researchers in 71 countries authored publications analyzed in this study. The top 10 countries accounted for 96.7% (3140 articles) of the total publications (Fig. 2). The United States had the highest number of publications at 1314 (40.5%) followed by China (25.1%) at 815 and India at 174 (5.4%) rounding out the top 3 counties by output. The publication productivity among the top 10 countries differed by as much as a factor of 14.9 (1st country versus the 10th country by rank).

### Publications by institutions for Folate receptor articles

**Fig. 3:**
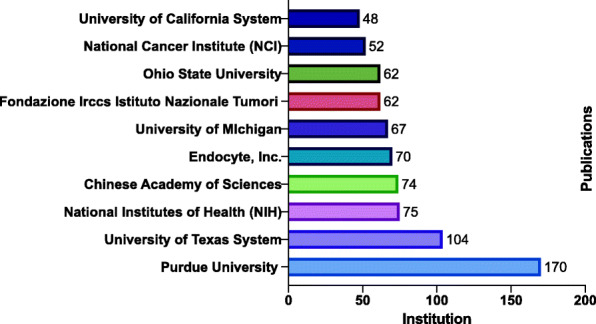
Publications for folate receptor research at the top 10 institutions

The total number of unique institutions represented in this study was 2351. The top 10 institutions accounted for 24.1% (754 articles) of the total number of publications (Fig. 3). Purdue University had the highest number of publications at 170 (5.2%) followed by the University of Texas System at 104 (3.2%) and the National Institutes of Health (NIH) at 75 (2.3%) to round out the top 3 counties by output. Of note, a privately held company was in 5th position at 70 research articles. The publication productivity among the top 10 institutions differed by as much as a factor of 3.5 (1st institution versus the 10th institution by rank).

### Publications by authors for Folate receptor articles

**Fig. 4:**
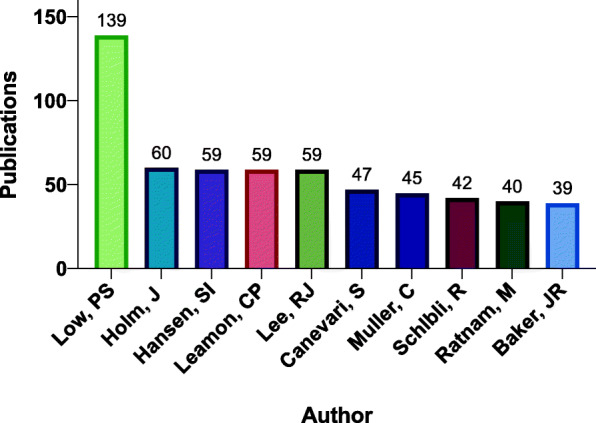
Publications for the top 10 authors in folate receptor research

The top 10 authors accounted for 18.1% (589) of the total number of research articles (Fig. 4). Low, P.S., had the highest number of publications at 139 (4.3%) followed by Holm, J. at 60 (1.8%) and Hansen, S.I., Lee, R.J., and Leamon, C.P tied at 59 (1.8%) by output. The publication productivity among the top 10 authors differed by as much as a factor of 3.6 and was consistent with institution affiliation data (1st author versus the 10th author by rank).

### Top 10 citations for Folate receptor articles

The top 10 research articles by total citations are listed in Table 2 [71] (Table follows the format established by Viana, et al). Also listed in 2 are citations per year (WoS does not provide standard deviation in their analytics for citations per year thus these values were calculated in Excel). These 10 articles have been cited 7270 times during the time frame of this study. The top cited article had a mean citation rate of 110 citations per year among the top 10 (Standard deviation). Mean citation rates differed by as much as a factor of 2. (1st paper versus the 10th paper by rank). Topics ranged from imaging (top 2 cited articles) to basic research articles regarding the distribution of folate receptor to therapies and other diagnostic assays. In fact, imaging and therapies were featured in 5 of the top 10 cited articles.

**Table 2:** Top 10 referenced publications by articles in the field of folate receptor or folate binding protein

Author	Title	Year	Total Citations	Citation Rate Index^a^
Kam et al.	Carbon nanotubes as multifunctional biological transporters and near-infrared agents for selective cancer cell destruction	2005	1667	119
Liong et al.	Multifunctional inorganic nanoparticles for imaging, targeting, and drug delivery	2008	1309	119
Varma et al.	GPI-anchored proteins are organized in submicron domains at the cell surface	1998	921	44
Weitman et al.	Distribution of the folate receptor GP38 in normal and malignant-cell lines and tissues	1992	893	33
van Dam et al.	Intraoperative tumor-specific fluorescence imaging in ovarian cancer by folate receptor-alpha targeting: first in-human results	2011	822	103
Ross et al.	Differential regulation of folate receptor isoforms in normal and malignant-tissues in-vivo and in established cell-lines – physiological and clinical implications	1994	754	30
Parker et al.	Folate receptor expression in carcinomas and normal tissues determined by a quantitative radioligand binding assay	2005	725	52
Kukowska-Latallo et al.	Nanoparticle targeting of anticancer drug improves therapeutic response in animal model of human epithelial cancer	2005	657	47
Smart et al.	A detergent-free method for purifying caveolae membrane from tissue-culture cells	1995	648	27
Kershaw et al.	A phase I study on adoptive immunotherapy using gene-modified T cells for ovarian cancer	2006	593	46

^a^Citation Rate Index mean = 62, standard deviation = 34.89

### Folate receptor articles related to Cancer, macrophages and imaging

**Fig. 5:**
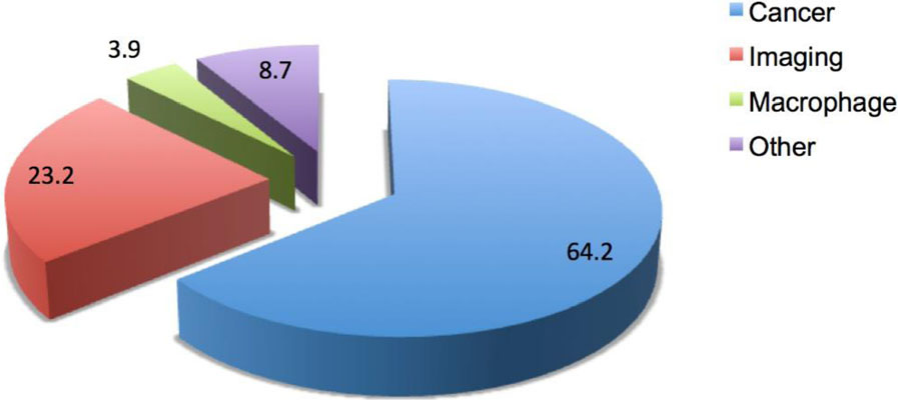
Percentage of folate receptor articles associated with cancer, macrophage and imaging keywords

**Fig. 6:**
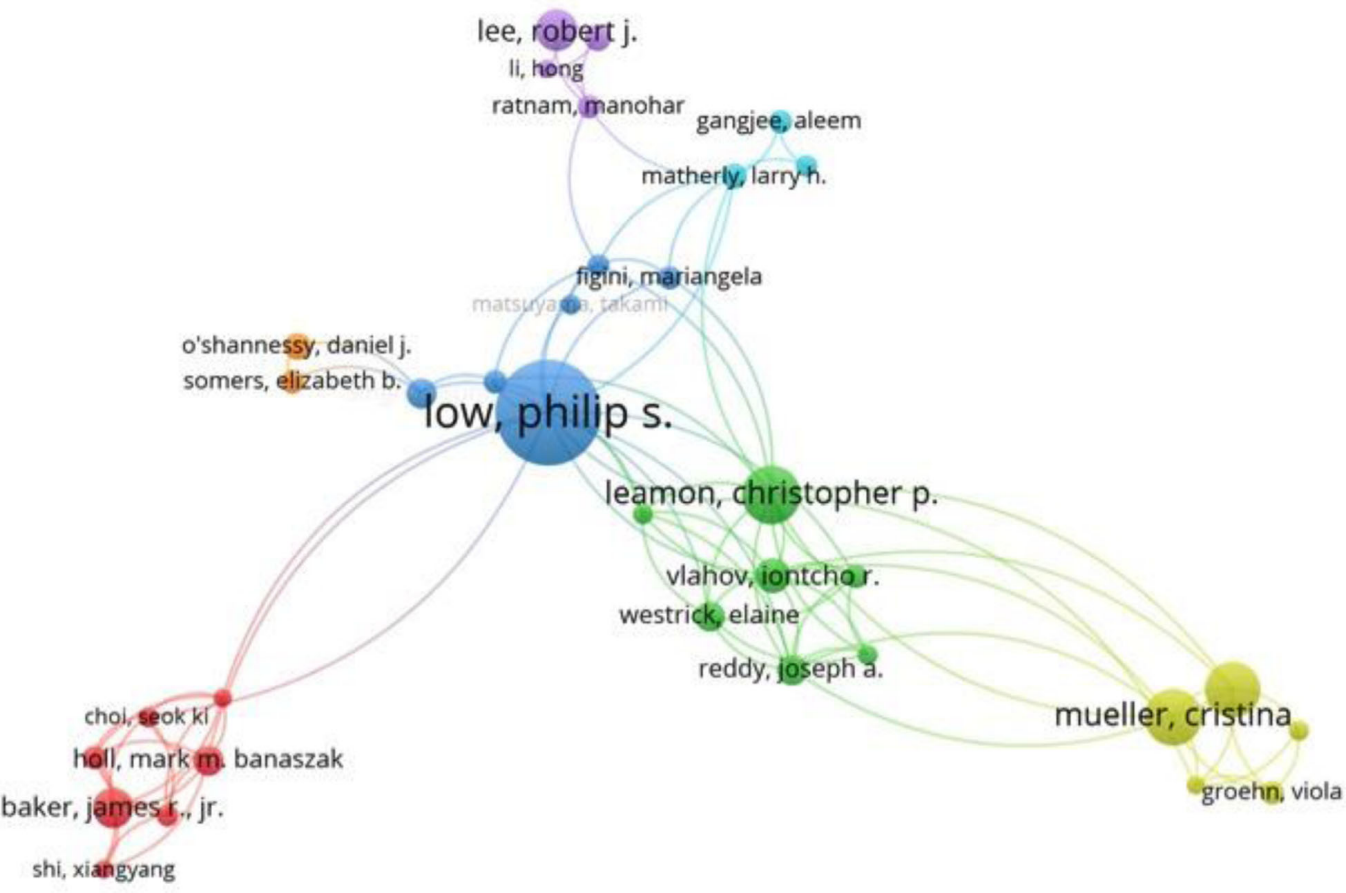
Term density map of folate receptor research in cancer

**Fig. 7:**
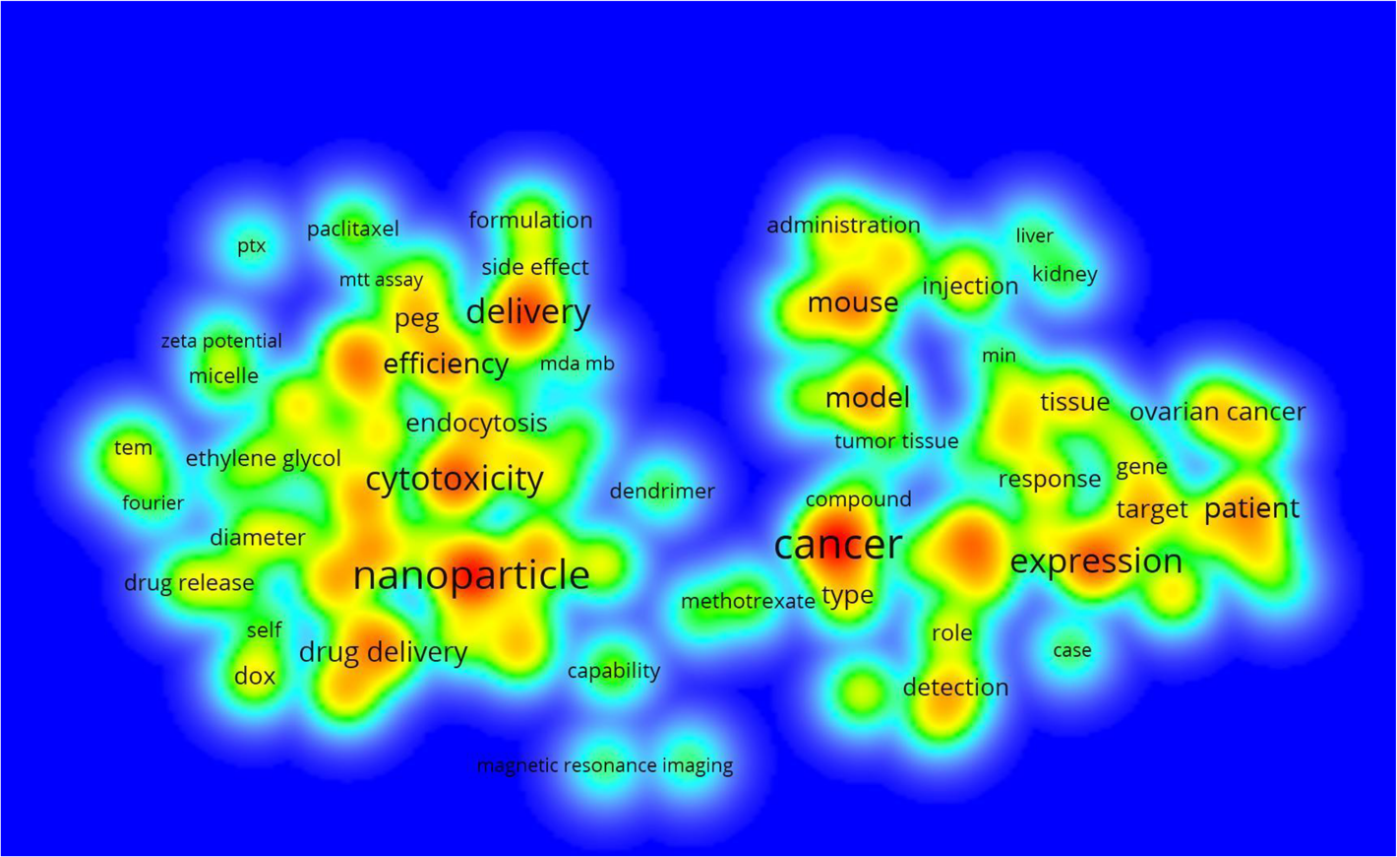
Term density map of folate receptor research in imaging

Since folate receptor is a well-known target for cancer therapies, modalities associated with cells of the immune system (macrophages) and imaging agents, a refined search (see methods section) was conducted. Search queries involving cancer, macrophage and imaging agents revealed 2085 articles (64.2%), 128 articles (3.9%) and 752 articles (23.2%), respectively, for each topical area. See Fig. 5. Bibliometric item intensity maps were constructed using VOSviewer version 1.6.11 [70] for both cancer and imaging (see Figs. 6 and 7).

### Co-author Bibliometric network

**Fig. 8:**
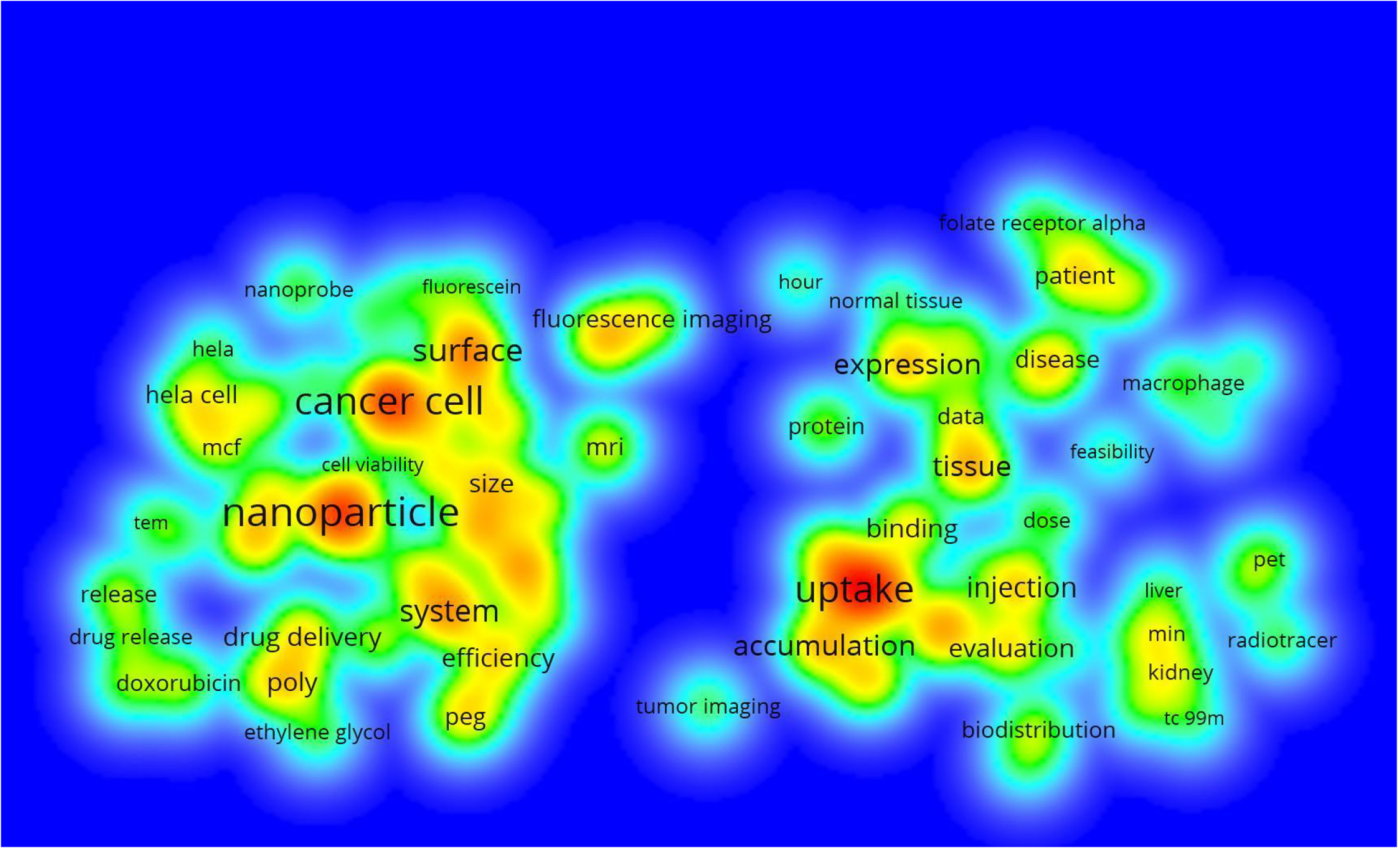
Co-authorship network map of publications in the field of folate receptor or folate binding protein research

A co-authorship bibliometric network map was constructed using VOSviewer version 1.6.11 [70]. Major co-authorship researcher nodes included: Low, P.S., Leamon, C, Mueller, C and Lee, R.J. and Baker, JR. See Fig. 8.

## Discussion

Folate receptor research publications increased considerably after the seminal work of Low and Leamon, which demonstrated uptake of conjugates via the folate receptor in receptor positive cancer cell lines [2]. The number of articles increased from 22 in 1991 to 263 in 2018 (the last full year studied). Although bibliometric studies of protein receptor ligands are limited, for comparison, a recent 25 year analysis of Sigma-1 receptor [72] yielded 1102 papers with 29,646 citations versus 3248 and 123,720 for folate receptor research. While the study of Sigma-1 receptor shows that article growth was not constant over time with large periods of stagnation, folate receptor research has experienced dramatic growth over roughly the same time frame with no periods of stagnation.

The large co-authorship nodes in the network map mirror several of the top 10 ten authors with Low occupying the central region, which is not surprising based on the historical progression of the field. Other prominent nodes included Mueller, Lee, Baker and Leamon. Given that folate receptor expression was found to be increased on cancer versus normal tissues, this feature provided a potential pathway for production of both therapeutic and diagnostic imaging agents. In fact, imaging in this early study played a key part in elucidation of folate mediated uptake of attached cargos [2]. Clinically, populations that will benefit from such targeted therapies need to be identified for treatment. This has been underscored by FDA guidance on the use of companion diagnostic for targeted therapies [73]. In addition to folate based MRI, CT, SPECT, PET and NIR imaging modalities [74], folate targeted agents are currently being explored for image-guided surgery for dubulkment of ovarian cancer tissue [75]. In our analysis, a folate guided surgery publication was the 5th most cited.

Moreover, our study detected an appreciable number of review articles (426 total) in addition to primary research articles that could be analyzed in future studies. A recent report by Blumel and Shniederman, based on inputs from the Conference of the International Society of Informetrics [76], proposes a broad agenda for the study of review articles. However, including such sizable numbers review articles within the framework of our current analyses would have been problematic. For instance, Ho [77] illustrates that review papers introduce bias in citation based analysis and that scholars must consider the purpose of the study and treat review papers distinctly to avoid this bias. Similar concerns have been earlier noted by Knottnerus [78]. There are also divergent definitions of what constitutes a review article including issues pointed out with WoS versus other databases noted by Ketcham [79].

The United States was the top article producing country followed by China and India. This is consistent with the output of Low (Purdue), Leamon (Endocyte), Lee (The Ohio State University), and the company Endoycte based in Purdue Research Park. There was a wide variation of publication productivity among the top 10 countries (differing by as much as a factor of 14.9 from the 1st country versus the 10th country by rank). Large state and federal (universities and institutes) and systems made up a large share of the top 10 institutions (e.g., Purdue, University of Texas, Chinese Academy of Sciences and NIH) with the notable exception of the private company Endocyte.

The top 10 cited articles revealed a high proportion of therapeutic and imaging related studies. Specifically, several works outline the use of folate-guided nanomaterials agents for detection and treatment of folate receptor positive cancers. Folate is easily conjugated to various nanomaterials and several high receptor expressing cancer cell lines exist for testing these nanoconjugates [72]. The field of nanomedicine, in general, also experienced rapid citation growth during this time frame [80]. An analysis of citation rates per time, Table 2, revealed that the top two citations by Kam et al., and Liong et al., were tied with 119 citations per number of years since publication. Van Dam et al., while in the fifth position, has an average citation rate of 103. Assessing the citation rate over time adds another layer of depth to the study of citations. Van der Pol et al. found a positive correlation between the quality and completeness of studies that adjusted for citation rates [81]. Hutchins et al. take it a step further and utilize a relative citation ratio whereby citation rates are divided by an expected citation rate that is a derivation from other articles in the same field within a peer comparison group [82]. This concept while not employed in this study, can be explored for further applications within the rich subfields of folate research.

Cancer was a key word associated with the majority of papers, followed by imaging and macrophages. Item density analyses of folate receptor subset areas yielded key topics including: nanoparticle, cytotoxicity, delivery, efficiency, expression, ovarian cancer, and fluorescence imaging, These areas were heavily represented in the top cited articles, especially articles associated with nanotechnology as previously outlined. In fact, the top two cited articles in folate receptor research were in nanotechnology. This information could be used to help guide further analyses in this area of research.

This study has limitations that are related to the exclusive use of Web of Science. As Zyoud et al., cites English is the indisputable language of science, but this in itself is a limitation for bibliometric studies as databases omit publications written in other languages [83]. Web of Science was chosen for this study because, as Chen states, its records are standardized and more consistent than competitors [84]. The authors acknowledge that different databases will return different items. However, as Mansoori noted, Web of Science offered better tools to refine and analyze searches than Scopus [85].

## Conclusions

Folate receptor research has led to the development of promising drug, imaging and other diagnostic schemes as well has had a profound impact on our understanding of receptor mediated cellular pathways. In this study, Web of Science was used to assess the global scientific production ranging from 1969-to June 28, 2019. Results illustrated a substantial increase in the cumulative volume of papers (264 per year for the last year studied). The US held the top metrics in publications, institutions and author output. The top 10 cited articles had an appreciable number of imaging and therapeutic applications, including nanotechnology. Given the different subareas of this dataset (e.g. imaging), there exists a rich source of data for further studies. Through bibliometric analysis, it should be possible to elucidate further interesting trends in these areas.

## Data Availability

The datasets used and/or analyzed during the current study are available from the corresponding author on reasonable request.
