# Correction to: A Bibliometric analysis of folate receptor research

**DOI:** 10.1186/s12885-020-07776-3

**Published:** 2021-01-14

**Authors:** Cari A. Didion, Walter A. Henne

**Affiliations:** Governors State University, 1 University Parkway, University Park, IL 60484 USA

**Correction to: BMC Cancer 20, 1109 (2020)**

**https://doi.org/10.1186/s12885-020-07607-5**

Following publication of the original article [1], the authors reported a typesetting error in the captions of Fig. 6 and Fig. 8. The two captions were mistakenly transposed. The correct figures and captions are given in this correction article.

Further to this, the equal contribution statement as been updated.

The original article [1] has been corrected.

**Fig. 6:**
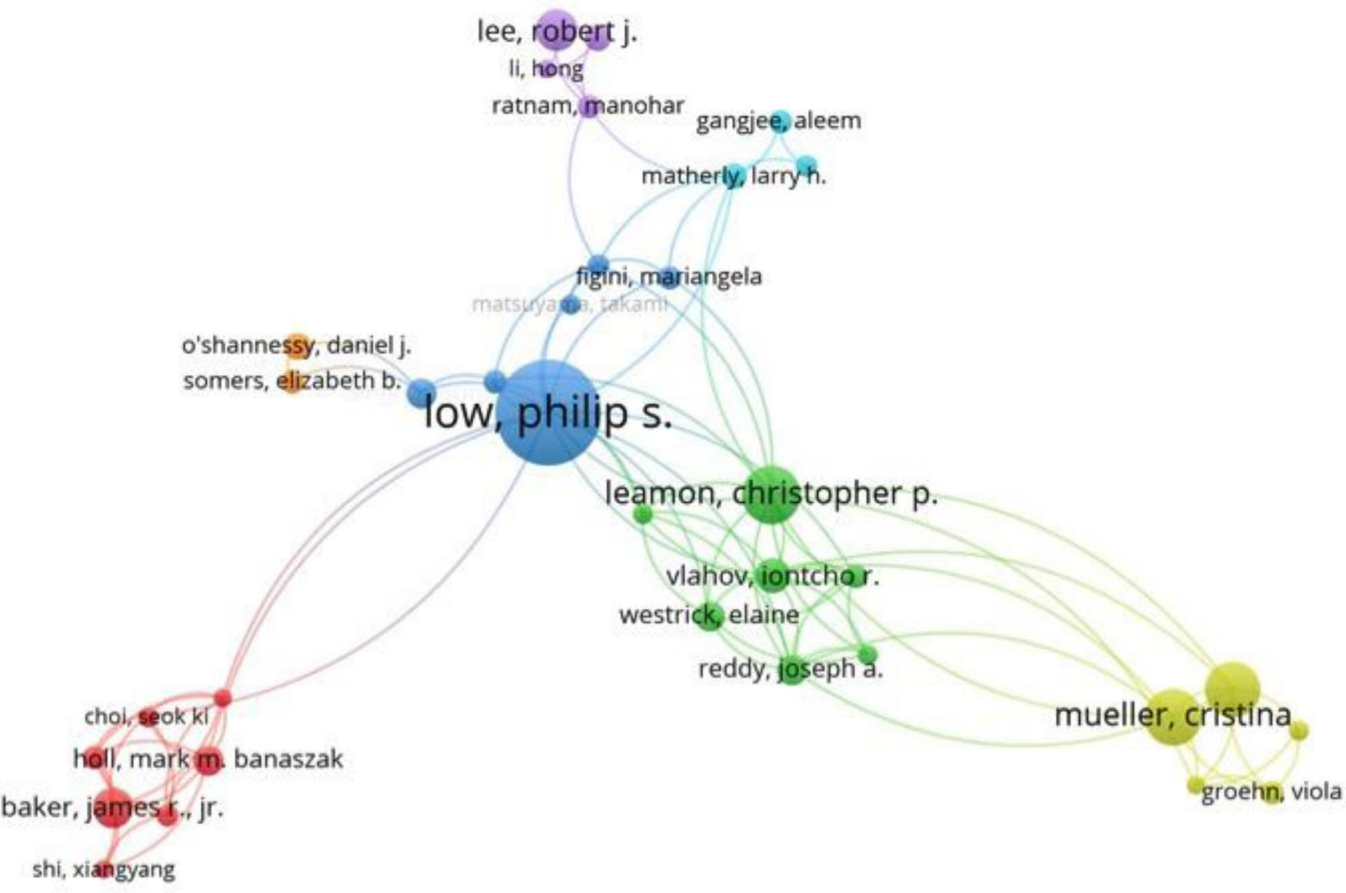
Co-authorship network map of publications in the field of folate receptor or folate binding protein research

**Fig. 8:**
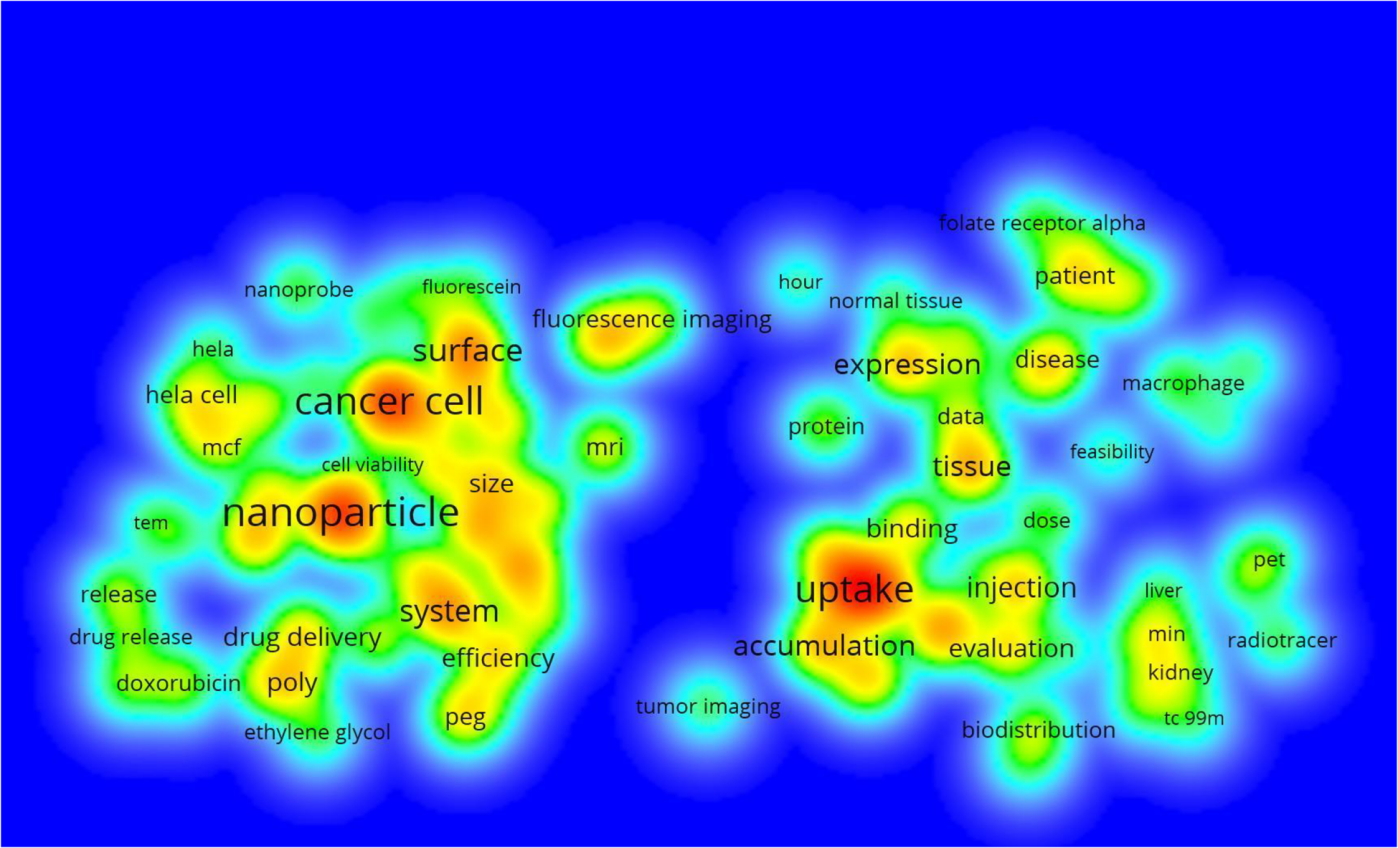
Term density map of folate receptor research in cancer
